# Ergonomic risk assessment for musculoskeletal disorders in office workers Turkish version, validity and reliability study

**DOI:** 10.7717/peerj.21318

**Published:** 2026-05-19

**Authors:** Erkan Erol, Halime Arıkan

**Affiliations:** Tokat Gaziosmanpaşa University, Department of Physiotherapy and Rehabilitation, Faculty of Health Sciences, Tokat, Turkey

**Keywords:** Ergonomic risk assessment for musculoskeletal disorders in office workers, Office worker, Validity, Reliability, Turkish version, Ergonomic risk

## Abstract

**Background:**

The Ergonomic Risk Assessment for Musculoskeletal Disorders in Office Workers (ERAMO) questionnaire is used by office workers, practitioners or researchers to screen for ergonomic risk factors in the office environment. The study aims to establish the validity and reliability of the Turkish version of ERAMO questionnaire among office workers.

**Methods:**

A total of 116 office workers, 67 women and 49 men, participated in the study. Reliability was assessed through test-retest reliability and internal consistency measures. Validity was evaluated by structural, content, and face validity analyses. Floor and ceiling effects were examined to provide a comprehensive evaluation. ERAMO’s correlation with pain intensity, New York Posture Rating (NYPR), and Short Form-36 (SF-36) was also examined.

**Results:**

ERAMO demonstrated high reliability, with an intraclass correlation coefficient (ICC) value of 0.802 and a Cronbach’s α of 0.890. Structural validity was affirmed through both exploratory factor analysis (EFA) and confirmatory factor analysis (CFA). EFA revealed four factors, accounting for 61.888% of the total variation. ERAMO’s total score exhibited no floor or ceiling effect. ERAMO exhibited correlations ranging from poor to moderate (−0.185 to −0.413) with pain intensity, NYPR, and SF-36 subscales.

**Conclusion:**

ERAMO has demonstrated high reliability and good validity. ERAMO can be used to evaluate ergonomic risks among Turkish-speaking office workers.

## Introduction

The majority of today’s jobs consist of office work, which typically involves prolonged use of computers and other electronic devices (Luttmann, Schmidt & Jäger, 2010). Although office work does not require significant physical strength, it often entails repetitive tasks and long periods of inactivity that can lead to musculoskeletal disorders (Wahlström, 2005).

Work-related musculoskeletal disorders are strongly associated with ergonomic risk factors, particularly poor posture, and mainly affect the neck, back, and shoulders (Guduru et al., 2022). Their prevalence is high: in developed countries, up to 70–80% of office workers report musculoskeletal disorders related complaints (Landau et al., 2012). Such figures highlight the magnitude of the problem.

The impact of musculoskeletal disorders extends beyond health outcomes. They contribute to lost working hours, decreased productivity, and increased work-related costs (Mianehsaz et al., 2022). The financial burden is substantial, with estimates indicating that musculoskeletal disorders account for approximately 1% of the gross domestic product in developed countries (Coluci, Alexandre & De Freitas Pedrini, 2012). Consequently, musculoskeletal disorders in office workers represent not only an important occupational health problem but also a significant socioeconomic concern. Musculoskeletal disorders among employees pose a significant burden for both workers and employers, and in modern society, musculoskeletal problems represent the most common type of work-related health issue (Demissie, Bayih & Demmelash, 2024). The prevalence of musculoskeletal disorders among office workers ranges from 20% to 90% (Ağar, Yeginoğlu & Kızıltan, 2025).

To prevent musculoskeletal disorders in office employees, it is necessary to identify occupational risk factors. For this purpose, numerous tools have been developed to assess work-related risks that may lead to musculoskeletal disorders. Recent studies have highlighted the importance of developing and validating ergonomic risk assessment tools across diverse populations and work settings. For example, Ağar, Berşe & Dirgar (2023) developed the Workplace Work Environment Ergonomics Scale for nurses in Turkey, demonstrating high reliability and construct validity. Similarly, Mazloumi et al. (2025) validated a comprehensive ergonomic risk assessment method for agricultural workers, incorporating environmental, organizational, and individual risk factors, with acceptable psychometric performance. Furthermore, Agostinelli et al. (2024) validated a computer vision–based tool for posture assessment in manufacturing environments, reflecting emerging technological approaches in ergonomics research. Collectively, these recent contributions underscore the global emphasis on robust ergonomic assessment instruments.

In Turkey, several ergonomic and musculoskeletal health questionnaires have been translated and validated, such as the Cornell Musculoskeletal Discomfort Questionnaire (Erdinc, Hot & Ozkaya, 2011) and the Nordic Musculoskeletal Questionnaire (Kahraman, Genç & Göz, 2016). These instruments primarily assess discomfort and musculoskeletal symptoms but do not comprehensively evaluate ergonomic risk factors in office environments. Therefore, despite the availability of certain tools, a gap remains in culturally adapted, validated instruments that specifically capture the multifactorial ergonomic risks relevant to office-based work.

Kluay-On & Chaikumarn (2022) developed the Ergonomic Risk Assessment for Musculoskeletal Disorders in Office Workers (ERAMO), a concise and user-friendly questionnaire designed to identify ergonomic risk factors in office environments. While the ERAMO has shown good validity and reliability in its original version, no Turkish adaptation has yet been developed or validated. Given the high prevalence of musculoskeletal disorders among Turkish office workers and the limitations of existing instruments, adapting ERAMO into Turkish is both timely and necessary.

The present study therefore aimed to translate ERAMO into Turkish, conduct cultural adaptation, and evaluate its psychometric properties—including validity and reliability—among Turkish-speaking office workers. By providing a standardized, validated tool, this work seeks to facilitate ergonomic risk screening in Turkish occupational health contexts and contribute to the prevention of work-related musculoskeletal disorders.

## Materials & Methods

Prior to the study, permission to adapt the ERAMO into Turkish was obtained from its original developers, Pimporn Kluay-On and Montakarn Chaikumarn. The criterion for inclusion in the study is being an employee of Tokat Gaziosmanpaşa University and an office worker.

A total of 116 office workers from Tokat Gaziosmanpaşa University participated in this study. The inclusion criterion was being a full-time office worker at the university. Participants were informed about the content of the study, which was approved by the Tokat Gaziosmanpaşa University Social and Human Sciences Research Ethics Committee (Date: 15.08.2023, session number: 13, decision number: 01-39). Written consent was obtained from the participants. The clinical trial registration number of the study is NCT06108700 (Date: 25/10/2023).

### Sample size calculation

In the test–retest reliability analysis, the anticipated reliability coefficient (*ρ*1) was set at 0.85, while the lowest acceptable level (*ρ*0) was defined as 0.75 (Andresen, 2000). With assumptions of α = 0.05 and *β* = 0.20 (power = 80%), the required sample size was calculated as 99 participants. For convergent validity, a correlation coefficient of *r* = 0.50 was adopted (Feise & Menke, 2001), suggesting a minimum of 23 participants per parameter. Given that four parameters were evaluated, the total required sample size was estimated at 92. As highlighted in the work of Arıkan et al. (2025) a target of 99 participants was therefore deemed sufficient to provide adequate statistical power for both reliability and validity analyses. Ultimately, 116 participants completed the baseline assessment, and 100 participants completed the retest.

### Translation and cultural adaptation procedure

1. Forward Translation

The original ERAMO was independently translated into Turkish by two bilingual experts, one physiotherapist and one linguist, to capture both clinical and linguistic perspectives.

2. Synthesis

The two Turkish translations were compared and synthesized into a single version through consensus, ensuring conceptual consistency and linguistic clarity.

3. Backward Translation

This synthesized Turkish version was back-translated into English by two sworn translators who were blinded to the original questionnaire, in order to verify accuracy.

4. Expert Committee Review

An expert committee reviewed all versions and the back-translation, confirming conceptual, semantic, and experiential equivalence with the original ERAMO.

5. Pre-testing (Pilot Study)

The pre-final Turkish version was tested on 30 office workers to assess clarity and comprehensibility. No further modifications were deemed necessary (Beaton et al., 2000).

### Measures

*Ergonomic Risk Assessment for Musculoskeletal Disorders in Office Workers (ERAMO):* ERAMO consists of 10 questions and three subsections. The first part evaluates individual factors. It includes three items assessing the history of neck and lower back pain and electronic device use. The second section, the physical factors section, contains five questions about working posture. The third section, psychosocial factors, has two items about mental fatigue (Kluay-On & Chaikumarn, 2022).

The ERAMO was originally developed as a 29-item instrument. During its initial psychometric evaluation, 17 items were removed due to low item-total correlations, resulting in a 12-item version. After exploratory factor analysis (EFA), two additional items with factor loadings below 0.6 were excluded, leading to the final 10-item version consisting of four factors (Kluay-On & Chaikumarn, 2022). In our study, the validated 10-item final version was used in its entirety.

*New York Posture Rating Chart (NYPR):* In the NYPR, posture changes that may occur in 13 different body parts are observed and scored. In the evaluation, five points are given if the person’s posture is correct, three points are given if it is moderately impaired, and one point is given if it is seriously impaired.

*Short Form 36 (SF-36):* The scale consists of 36 questions and enables the evaluation of eight sub-dimensions. The subscales of the scale are physical function, social function, role limitations due to physical functions, role limitations due to emotional problems, mental health, energy, pain and general perception of health. The Turkish validity and reliability study of the scale was conducted by Kocyigit et al. (2003).

### Statistical analysis

Statistical analyses were conducted using Social Sciences Statistical Package SPSS version 22.0 (IBM Corp., Armonk, NY, USA). The results are presented as mean with standard deviation, median, or percentages. The distribution of the data was examined with the Kolmogorov–Smirnov test. To determine the reliability of the ERAMO, both internal consistency and test–retest evaluations were performed. Test–retest reliability was quantified using the intraclass correlation coefficient (ICC), whereas Cronbach’s α was employed to assess internal consistency. Reliability was considered high when ICC values exceeded 0.80 (Snyder et al., 2012). Cronbach’s α value of 0.70 and above for a newly developed scale and 0.80 and above for an established scale is acceptable (Zinbarg et al., 2005). EFA and confirmatory factor analysis (CFA) were conducted to assess the validity of ERAMO. Additionally, Pearson correlation analysis was employed to determine the correlation between ERAMO and continuous variables such as pain intensity, NYPR, and SF-36 scores. Correlations between 0.00−0.20, 0.21−0.40, 0.41−0.60, 0.61−0.80, and 0.81−1.00 correspond to poor, weak, moderate, high, and excellent correlations, respectively (Feise & Menke, 2001). CFA was evaluated by calculating the Minimum Fit Function Chi-Square (*χ*^2^), Degrees of Freedom (df), Standardized Root Mean Square Residual (SRMR), Comparative Fit Index (CFI), Goodness-of-Fit Index (GFI), Non-Normed Fit Index (NNFI), and Root Mean Square Error of Approximation (RMSEA). The fit indices of ERAMO were presented together with the acceptable and excellent fit values.

## Results

A total of 116 office workers participated in this study. The average age of the individuals was 40.00 ± 8.00, with 67 (57.8%) females and 49 (42.2%) males. The demographic characteristics of the individuals are presented in Table 1.

### Reliability

**Figure 1:**
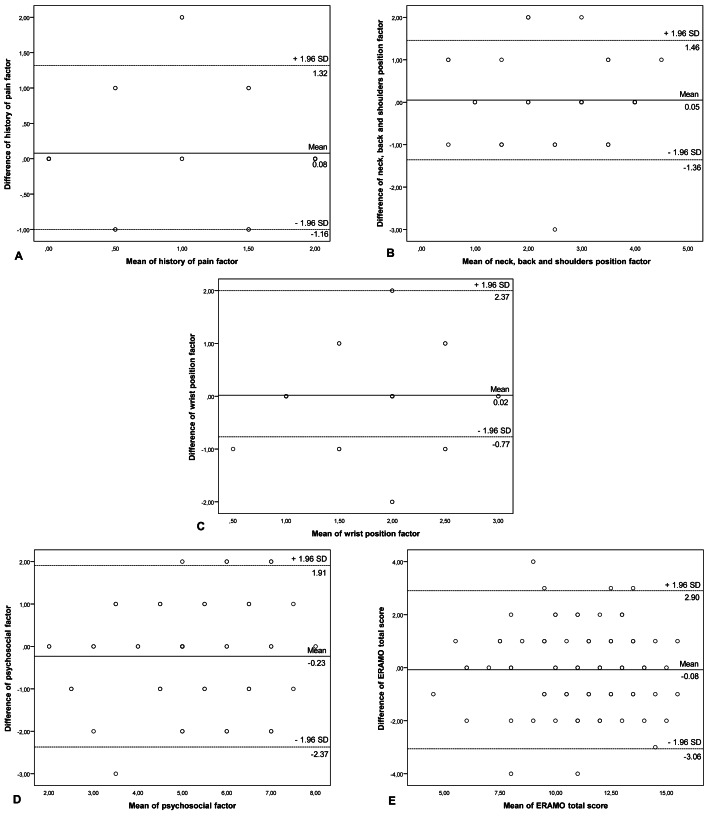
Bland-Altman plots for ERAMO test–retest reliability. The central line represents the mean differences between the first measurement and the second measurement; the upper and lower dotted lines represent the upper and lower 95% limits of agreement (Mean Differences ± 1.96 standard Deviation of the differences), respectively. (A) test-retest of the history of pain factor, (B) test-retest of neck, back and shoulder positions factor, (C) test-retest of wrist position factor, (D) test-retest of psychosocial factor, and (E) test-retest of the ERAMO total score.

Cronbach’s α for the total ERAMO score was 0.890, indicating high internal consistency. Subscale Cronbach’s α values ranged from 0.662 (wrist position factor) to 0.890 (neck, back and shoulders position factor). Although these subscale values are slightly lower than the total score, they remain within the acceptable range for established instruments. Test–retest reliability analysis with a 7-day interval yielded an ICC of 0.802 for the total score, 0.737 for history of pain factor, 0.802 for neck, back and shoulders position factor, 0.495 for wrist position and 0.696 for psychosocial factors, suggesting good reliability overall and moderate stability in subscales. The SEM and MDC for the ERAMO total score were 1.07 and 2.97, respectively (Table 2). Bland-Altman plots further confirmed the test-retest reliability of ERAMO (Fig. 1). The mean scores of ERAMO items corrected item-total correlations, and Cronbach’s α results in case of item deletion are presented in Table 3. This table showed that each item should be included in the Turkish version of ERAMO.

**Table 1:** Characteristics of individuals.

	**Test group (*n* = 116)**	**Retest group (*n* = 100)**
	**Mean ± SD**	
**Age**	40.00 ± 8.00	39.00 ± 8.00
**Weight**	72.00 ± 14.00	71.00 ± 13.00
**Length**	1.68 ± 0.09	1.67 ± 0.08
**BMI**	25.44 ± 3.74	25.15 ± 3.67
	**n (%)**	
**Gender**		
Female	67 (57.8)	62 (62.0)
Male	49 (42.2)	38 (38.0)
**Marital status**		
Single	36 (31.0)	35 (35.0)
Married	80 (69.0)	65 (65.0)
**Education history**		
Primary school	0 (0)	0
Middle school	0 (0)	0
High school	0 (0)	5 (5.0)
Associate degree	8 (6.9)	27 (27.0)
Bachelor’s degree or above	108 (93.1)	68 (68.0)
**Smoking**		
Yes	26 (22.4)	21 (21.0)
No	90 (77.6)	79 (79.0)
**Alcohol use**		
Yes	11 (9.5)	9 (9.0)
No	105 (90.5)	91 (91.0)

**Table 2:** Reliability of the ERAMO questionnaire (*n* = 100).

	**Baseline Mean ± SD**	**Retest Mean ± SD**	***p***	**Test-retest (ICC**_**2,1**_ ** and 95% CI)**	**SEM**	**MDC**	**Internal consistency (Cronbach’s α)**
**ERAMO total score**	11.13 ± 2.39	11.21 ± 2.43	0.599	0.802 (0.719–0.862)	1.07	2.97	0.890
**History of pain factor**	1.05 ± 0.85	0.97 ± 0.89	0.208	0.737 (0.633–0.815)	0.45	1.27	0.849
**Neck, back and shoulders position factor**	3.08 ± 1.13	3.03 ± 1.14	0.487	0.802 (0.719–0.862)	0.51	1.40	0.890
**Wrist position factor**	1.93 ± 0.83	1.91 ± 0.77	0.804	0.495 (0.332–0.630)	0.57	1.58	0.662
**Psychosocial factor**	5.07 ± 1.37	5.30 ± 1.42	0.037	0.696 (0.580–0.785)	0.77	2.14	0.821

**Table 3:** Corrected item-total correlation of the ERAMO questionnaire (*n* = 116).

	**Mean**	**SD**	**Corrected item-total correlation**	**Cronbach’s α if the item deleted**
1. In your life time, have you had back pain caused by working, with symptoms lasting longer than 24 hours? *Yaşam*ı*n*ı*z boyunca, çal*ı*şmaktan kaynaklanan ve 24 saatten uzun süren bel ağr*ı*n*ı*z oldu mu?*	0.49	0.50	0.048	0.404
2. In your life time, have you had neck pain caused by working, with symptoms lasting longer than 24 hours? *Yaşam*ı*n*ı*z boyunca, çal*ı*şmaktan kaynaklanan ve 24 saatten uzun süren boyun ağr*ı*n*ı*z oldu mu?*	0.59	0.49	0.059	0.401
3. When you used the former device, what is your head or neck position? *Masaüstü bilgisayar, dizüstü bilgisayar, tablet veya ak*ı*ll*ı* telefon gibi elektronik cihazlar*ı* kullan*ı*rken baş-boyun pozisyonunuz nas*ı*ld*ı*r?*	0.75	0.44	0.385	0.307
4. What is your neck position during sitting at work? *İş yerinde otururken boyun pozisyonunuz nas*ı*ld*ı*r?*	0.71	0.46	0.429	0.288
5. What is your trunk position during sitting at work? *İş yerinde otururken gövde pozisyonunuz nas*ı*ld*ı*r?*	0.90	0.31	0.194	0.371
6. What is the position of your shoulders and arms during typing? *Bilgisayarda yaz*ı* yazarken omuzlar*ı*n*ı*z*ı*n ve kollar*ı*n*ı*z*ı*n pozisyonu nas*ı*ld*ı*r?*	0.64	0.48	0.276	0.333
7. What is the position of your wrists during typing? *Bilgisayarda yaz*ı* yazarken bileklerinizin pozisyonu nas*ı*ld*ı*r?*	1.21	0.84	0.083	0.414
8. Are your wrists entrapped during typing? *Bilgisayarda yaz*ı* yazarken bilekleriniz s*ı*k*ı*ş*ı*yor mu?*	0.74	0.44	−0.278	0.484
9. Do you find your work breaks sufficient? *Çal*ı*şma molalar*ı*n*ı*z*ı* yeterli buluyor musunuz?*	2.00	0.84	0.114	0.397
10. You feel mentally exhausted. *Zihinsel olarak yorgun hissediyorsunuz.*	2.95	0.95	0.363	0.238

**Table 4:** Factor loading of the ERAMO questionnaire (*n* = 116).

**Items**	**History of pain factor**	**Neck, back and shoulders position factor**	**Wrist position factor**	**Psychosocial factor**
**Item 1**	0.846			
**Item 2**	0.771			
**Item 3**		0.714		
**Item 4**		0.823		
**Item 5**		0.660		
**Item 6**		0.465		
**Item 7**			0.689	
**Item 8**			−0.771	
**Item 9**				0.884
**Item 10**				0.636
**Percent variance (%)**	14.497	35.066	48.729	61.888

### Validity

**Figure 2:**
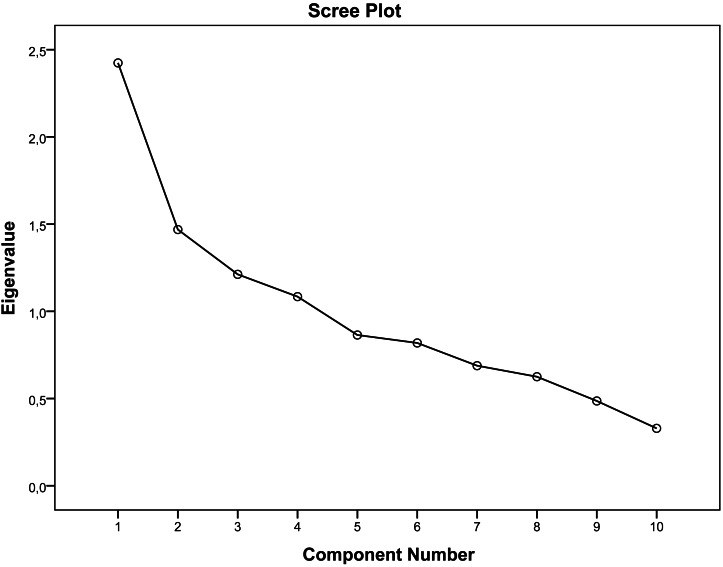
Scree plot of factor analysis for the Turkish ERAMO.

EFA supported a four-factor solution, explaining 64% of the variance. The Kaiser–Meyer–Olkin value was 0.624, and Bartlett’s test of sphericity was significant (*χ*^2^ = 164.004, *p* < 0.001). Item 6 loaded onto factor 2 instead of factor 3, differing from the original version (Table 4). The scree plot further substantiated the presence of the 4-factor structure (Fig. 2). CFA confirmed good model fit (*χ*^2^/*df* = 29.17/29 (1.01); SRMR = 0.069; CFI = 1.00; GFI = 0.95; NNFI = 1.00; RMSEA = 0.00) (Table 5).

**Table 5:** Goodness-of-fit indices for ERAMO questionnaire factor solution obtained by confirmatory factor analysis (*n* = 116).

**x** ^**2**^ **(df)**	**SRMR**	**CFI**	**GFI**	**NNFI**	**RMSEA**
29.17 (29)	0.069	1.00	0.95	1.00	0.00

**Notes.**

x^2^ Minimum Fit Function Chi-Square df Degrees of Freedom SRMR Standardized Root Mean Square Residual CFI Comparative Fit Index GFI Goodness-of-fit Index NNFI Non-normed Fit Index RMSEA Root Mean Square Error of Approximation

### Correlations with external measures

A poor correlation was observed between ERAMO and NYPR (−0.185). Weak correlations were noted between ERAMO and pain intensity (0.276), physical functioning (−0.373), role-physical (−0.327), role-emotional (−0.335), mental health (−0.363), social functioning (−0.366), bodily pain (−0.331), and general health (−0.283). A moderate correlation was identified between ERAMO and vitality (−0.413). ERAMO factors such as history of pain and wrist position were not associated with any outcome measures. Neck, back and shoulders position, and psychosocial factors showed weak correlations with outcome measures (Table 6).

**Table 6:** ERAMO questionnaire correlations with the pain intensity, NYPR and SF-36 scores (*n* = 116).

	**ERAMO total score**	**History of pian factor**	**Neck, back and shoulders position factor**	**Wrist position factor**	**Psychosocial factor**
**Pain intensity**	0.276^**^	0.103	0.296^**^	−0.127	0.234^*^
**NYPR**	−0.185^*^	−0.014	−0.248^**^	−0.134	−0.026
**Physical functioning**	−0.373^**^	−0.002	−0.217^*^	−0.145	−0.365^**^
**Role-physical**	−0.327^**^	−0.008	−0.212^*^	−0.010	−0.376^**^
**Role-emotional**	−0.335^**^	−0.124	−0.294^*^	0.094	−0.304^**^
**Vitality**	−0.413^**^	−0.166	−0.233^*^	−0.031	−0.381^**^
**Mental health**	−0.363^**^	−0.147	−0.171	−0.031	−0.367^**^
**Social functioning**	−0.366^**^	−0.169	−0.246^**^	−0.034	−0.290^**^
**Bodily pain**	−0.331^**^	−0.085	−0.244^**^	0.000	−0.302^**^
**General health**	−0.283^**^	−0.106	−0.098	0.010	−0.334^**^

**Notes.**

** *p* < 0.01.

* *p* < 0.05.

ERAMO Ergonomic Risk Assessment for Musculoskeletal Disorders in Office Workers Questionnaire NYPR New York Posture Rating Chart SF-36 Shot Form-36

### Ceiling effects

No floor or ceiling effects were detected for the total ERAMO score. However, ceiling effects were observed in the history of pain; neck, back and shoulders position; and wrist position factors, indicating reduced sensitivity in participants with generally favorable ergonomic conditions. The history of pain factor also showed a floor effect (Table 7).

**Table 7:** Floor and ceiling effects of the ERAMO questionnaire (*n* = 116).

	**Mean ± SD**	**Median (Min–Max)**	**Floor effect n (%)**	**Ceiling effect n (%)**
**ERAMO total score**	11.00 ± 2.00	11.00 (4.00–16.00)	0 (0)	0 (0)
**History of pain factor**	1.09 ± 0.83	1.00 (0.00–2.00)	35 (30.2)	45 (38.8)
**Neck, back and shoulders position factor**	2.99 ± 1.18	3.00 (0.00–4.00)	3 (2.6)	53 (45.7)
**Wrist position factor**	1.95 ± 0.83	2.00 (0.00–3.00)	1 (0.9)	36 (31.0)
**Psychosocial factor**	4.95 ± 1.44	5.00 (2.00–8.00)	10 (8.6)	4 (3.4)

## Discussion

This study aimed to translate ERAMO into Turkish, culturally adapt it, and assess its reliability and validity among office workers. Psychometric analyses revealed that ERAMO is a valid and reliable measurement tool for Turkish-speaking office workers.

Following the internal consistency evaluation of ERAMO, the original development study reported a Cronbach’s α of approximately 0.6, slightly below the acceptable threshold of 0.70, and ICC values only for earlier developmental versions (29-item and 12-item forms; ICC = 0.79 for the 12-item form), but not for the final 10-item version. In contrast, in the current study, Cronbach’s α ranged between 0.662 and 0.890 for the total score and subscales, indicating high internal consistency. The ICC values ranged from 0.802 for the total score to 0.495–0.737 for the subscales, reflecting good overall temporal stability. Although the subscale reliability values were moderate, this finding is not unexpected. Subscales often contain fewer items and measure more specific aspects of a construct, which can result in lower reliability compared to the total score. In this context, the high reliability of the total ERAMO score indicates that the instrument as a whole is robust, while the moderate subscale values reflect the inherent variability of multidimensional constructs. Therefore, the Turkish version of ERAMO can still be considered reproducible and suitable for practical use, particularly when the total score is prioritized.

In the Turkish version of ERAMO, Cronbach’s α if item deleted values indicated that all ten items should be retained in the questionnaire. Removing any item from the questionnaire resulted in a value lower than the overall Cronbach’s α. The SEM value corresponds to 9.75% of the ERAMO average and 6.30% of the maximum possible value. The MDC represents 27.05% of the ERAMO average and 15.71% of the maximum score. For future version studies, it is advisable to calculate these values to facilitate comparisons in terms of repeatability.

Structural validity was supported by both EFA and CFA. Original ERAMO demonstrated a 4-factor structure in the EFA, which CFA further confirmed. Accordingly, items 1 and 2 loaded onto the “history of pain”; items 3, 4, and 5 loaded onto the “neck and back position”; items 6, 7, and 8 loaded onto the “upper extremities position”; and items 9 and 10 loaded onto the “psychosocial” factors. Following the CFA, it was observed that the goodness-of-fit indices values were entirely satisfactory (Kluay-On & Chaikumarn, 2022).

The Turkish version exhibited a four-factor structure similar to the original version, except for item 6, which loaded onto a different factor. The renaming of factors in the Turkish ERAMO was conducted to better capture the conceptual meaning of items within this cultural context. Although the original factor structure was largely preserved, slight variations in item loadings necessitated adjustments in factor labels. Specifically, the “neck, back, and shoulders position” and “wrist position” factors more accurately reflect the ergonomic behaviors assessed in the Turkish sample. Such minor modifications are common in cross-cultural adaptations of psychometric instruments and do not compromise the structural validity of the scale.

Regarding correlations with external measures, ERAMO demonstrated poor-to-moderate relationships with pain intensity, posture (NYPR), and SF-36 subscales. At first glance, these findings may seem to limit its construct validity. However, they should be interpreted within the broader methodological and conceptual framework. Musculoskeletal disorders and quality of life are multifactorial phenomena influenced not only by ergonomics but also by psychosocial, organizational, and individual factors. In this context, the weak-to-moderate correlations underscore the specificity of ERAMO in capturing ergonomic risk factors rather than broader health outcomes, which should be considered a strength of the instrument rather than a limitation.

The ceiling effect observed in certain subscales (history of pain; neck, back and shoulders position; and wrist position factors) represents another methodological consideration. While the total ERAMO score did not exhibit ceiling or floor effects, the presence of ceiling effects in subscales suggests that these domains may have limited sensitivity in detecting subtle changes among office workers with generally good ergonomic conditions. For practical applications, we therefore recommend prioritizing the total score, while interpreting subscale scores with caution. Another limitation of this study is that all participants were recruited from a single university, which may restrict the generalizability of the findings to broader office worker populations.

Taken together, these findings support the reliability and validity of the Turkish ERAMO, while also highlighting areas that warrant cautious interpretation. The instrument provides a practical, culturally relevant tool for screening ergonomic risks among office workers in Turkey. Beyond its methodological contributions, the Turkish ERAMO has potential clinical and occupational benefits, such as guiding workplace interventions, informing ergonomic education, and contributing to early prevention strategies.

## Conclusions

The Turkish version of the ERAMO is a valid and reliable instrument for assessing ergonomic risk factors among Turkish-speaking office workers. Its brevity, clarity, and ease of application make it highly suitable for both clinical and research contexts. More importantly, by identifying ergonomic risks in the workplace, ERAMO contributes to the early detection and prevention of work-related musculoskeletal disorders. The use of ERAMO in occupational settings has the potential to enhance occupational performance, reduce disability, and promote health through ergonomic interventions tailored to individual and environmental needs.

##  Supplemental Information

10.7717/peerj.21318/supp-1 Supplemental Information 1 Raw data

## Additional Information and Declarations

### Data Availability

The following information was supplied regarding data availability: The raw data are available in the Supplemental File.
